# Progress in Psychiatry

**Published:** 1903-08-01

**Authors:** 


### Progress in Psychiatry.

(*Continued from Page* 298.)

**Morphinomania and its Treatment by Sudden Withdrawal of the Drug.**—Dr. Margaret Halleck, of the New York State Reformatory for Women, states[8] that there need be no risk for the patient in the treatment of morphinomania by "sudden withdrawal" of the drug if proper precautions are taken. "As each patient came under care I endeavoured to win her confidence . . . I told her frankly that no morphia would be administered, but that she would not be allowed to suffer much." "Suggestion" was freely used and personal interest shown in every case. Five cases are recorded, of which cases Nos. 1 and 2 were smokers of opium for from three to ten years, case No. 3 had taken morphia for ten years by the mouth, while cases Nos. 4 and 5 had taken morphia by the mouth as well as hypodermically for seven years. An abstract of cases Nos. 1 and 4 is appended. Case No. 1 was a woman aged 24 years. The morphia habit originated when she was under treatment for a broken leg. She now takes 6 to 10 grains of morphia daily besides smoking a "dollar's worth of crude opium." On admission to the reformatory she was suffering from vomiting, cold perspiration, extreme nervousness, and aching all over, while the pupils were dilated. She was put to bed and given the following medicine hypodermically, viz., strychnine sulphate $\frac{1}{60}$th of a grain, hyoscine hydrobromate $\frac{1}{200}$th of a grain, codeine sulphate $\frac{1}{4}$ of a grain. This was given nightly with the suggestion that she would sleep well. Sleep improved day by day and she was free from pain and vomiting. Steady improvement showed itself during the first week. The injection was then reduced to strychnine only ($\frac{1}{60}$th of a grain) for another week, and then all medication was stopped. Her weight on admission was 99 lbs. In six months she had gained 30 lbs. She remained for a year quite cured, and then left the institution. So far there has been no relapse. Case No. 4 was a woman, aged 24 years, who was taught the morphia habit by an older woman. She had taken morphia hypodermically for seven years, the present daily dose being 50 to 60 grains. In the spring of 1902 she entered an institution to be cured of the habit, and after improvement she relapsed three separate times. On admission to the Reformatory she was very emaciated (weight 90 lbs.), nervous, with slow pulse, dilated pupils, and constipation. She was put to bed and given one tablet each of hyoscine and codeine at noon, and the same quantity at 8 p.m. The medicines were slightly varied in dosage according to the symptoms present, and reduced in quantity till in 17 days (December 11th) she was taking only one strychnine tablet. The latter has since then been discontinued. "She now eats and sleeps well, and has gained weight," her present weight being $114\frac{1}{2}$ lbs., a gain of over 20 lbs. in six weeks. All the cases, says Dr. Halleck, seem to have lost their craving, and appear bright, cheerful, and well-nourished. "Strychnine, hyoscine, and codeine form a perfect antidotal combination, which works quickly." Morphia, as is well known, depresses the nervous system, reduces metabolism, paralyses the vaso-motor centres, and lessens all secretions. Strychnine, on the other hand, stimulates all functions and increases the appetite, while the nausea, restlessness, and insomnia are combated by the hyoscine and the codeine.

**Types of Opium-eaters.**—Smith Ely Jelliffe,[9] neurologist to the City Hospital, New York, finds that the opium habit, like alcoholism, produces different results in those who indulge in it. The drinker of beer, gin, port, and Burgundy reacts differently to these liquors, and the opium-eater varies correspondingly to a smaller extent. There are three classes of opium-takers—viz. (*a*) those who take the drug in the form of a medicinal preparation (laudanum, paregoric, extract of opium) by the mouth, rectum, or vagina; (*b*) those who smoke it and inhale the vapour into their lungs, a class which includes the largest number of habitués; and (*c*) those who take the alkaloid morphia by the mouth or hypodermically. The sale of opium in the State of Vermont, with its population of 187,000 souls, is estimated to be over $3\frac{1}{4}$ millions of pharmacopœal doses of opium every month, a quantity equivalent to a daily dose of 1 grain for every male and female over the age of 21 years. The opium-taker, like other individuals, comprises persons of varied temperaments. The fundamental characteristic is a desire or craving for exhilaration and excitement, comparable to that which has led some to the consumption of coca, cannabis indica, and mescal. The opium habitué, like the dipsomaniac, is one in whom the craving for "intense" states of consciousness is over-developed, and who is also lacking in self-control. A large class includes those who become addicted to it to obtain alleviation from pain or bodily suffering or mental distress. The following two psychological types are met with. The first of these is like the dipsomaniac, the patient being the victim of the habit only at recurrent periods (periods of debauch lasting three or four days), after which a healthy interval of several months is passed during which he is sober and free from craving or taking of the drug. The second type includes the steady smoker who attends to his daily work but spends two or three hours in the evening in smoking opium, the habit being indulged in four or five evenings a week. "For a number of years he continues thus, and does not suffer to any great extent. When sudden intercurrent disease comes, the strain tells, and pneumonia, typhoid fever, dysentery, and gastritis prove fatal with greater frequency than in the non-habituated." Treatment includes, first, substitution of a different set of ideas to resist or overcome the craving, which may be done by mental "suggestion;" and secondly, the administration of drugs to satisfy partially the craving and overcome the distress consequent on enforced deprivation of the opium or morphia. These two factors judiciously combined will prove effective even in the most intractable cases. The sedative drug recommended by Jelliffe is the bromide of sodium in large doses to produce a slight feeling of drowsiness, supplemented by hyoscine and heroin. Heroin especially tends to abolish the craving for morphia, as shown by Morel Levallée. A report of four cases is appended by the author showing the satisfactory results of treatment.

**General Paralysis of the Insane: Clinical Statistics.**—Soukhanoff and Gannouchine of Moscow have published[10] records of their investigation of all cases of general paralysis of the insane admitted to the Psychiatric Clinic of Moscow during the period from November, 1887, to January 1901. The total number of general paralytics was 682 out of 3,916 patients of all classes. Of these 590 were males and 92 females. During the last four years of the period—viz., 1897 to 1900 inclusive—the proportion of general paralytics among males has risen higher than that among females. The age at which the disease occurs was ascertained to be as follows:—

| Age. | Percentage of males. | Percentage of females. |
|---|---|---|
| Below 20 years | 0·17 | 3·26 |
| From 20 to 25 years | 0·69 | 5·44 |
| „ 26 to 30 „ | 11·53 | 9·78 |
| „ 31 to 35 „ | 26·16 | 26·09 |
| „ 36 to 40 „ | 31·33 | 23 91 |
| „ 41 to 45 „ | 15·32 | 13·04 |
| „ 46 to 50 „ | 8·95 | 9·78 |
| „ 51 to 55 „ | 3·44 | 6·52 |
| „ 56 to 60 „ | 1·89 | 2·18 |
| Above 60 years | 0 52 | 0·00 |

Three cases of juvenile general paralysis were met with, viz., a girl aged 16 years, the subject of inherited or congenital syphilis; a girl, aged 20 years, whose father was a drunkard, though the causation of the general paralysis in this case was doubtful; and a boy, aged 19 years, whose father was a drunkard and whose mother was insane. Of the male general paralytics 79 per cent. were married, 19 per cent. were single, and 2 per cent. were widowers. Of the female general paralytics 65 per cent. were married, 13 per cent. were single, and 22 per cent. were widows. General paralysis was found to be less frequent among the agricultural population than among town dwellers. Syphilis as an ætiological factor could be traced with certainty in 61½ per cent. of males and 21 per cent. of females. The presence of syphilis was "probable" in another 9 per cent. of males and 25 per cent. of females. In a further 9 per cent. of males and 16 per cent. of females the occurrence of syphilis was a "possibility." Syphilis, however, could be excluded with certainty in 20 per cent. of males and 39 per cent. of females. Allowing a liberal margin therefore for "doubtful" and "possible" cases, syphilis as an ætiological factor was traceable in four-fifths of all male and three-fifths of all female cases, the remaining proportions being of non-syphilitic origin. The interval between the date of syphilitic infection and the onset of general paralysis was ascertained correctly in 265 male cases, with the following results: 5·7 per cent. of cases occurred within five years of the date of infection, 37 per cent. occurred in from 6 to 10 years, 36 per cent. occurred in from 11 to 15 years, 16 per cent. occurred in from 16 to 20 years, and 5 per cent. occurred after more than 20 years from the date of infection. The shortest period was three years, as observed in three cases. Alcoholic intemperance was traceable in 63 per cent. of males and 19 per cent. of females, while 14 per cent. of males and 19 per cent. of females were found to be moderate drinkers. Of the psychological types of general paralysis it was found that dementia was the predominant type in 50 per cent. of cases, maniacal excitement prevailed in 34 per cent., melancholic depression in 6 per cent., while the rest presented marked hypochondriacal and paranoid features. The predominantly demented type, add the authors, seems to be increasing year by year in both males and females. Inequality of the pupils was observed in 73 per cent. of males and 66 per cent. of females, the pupils being equal in the remainder. As regards mobility of the pupils the reactions were sluggish or absent in 82 per cent. of males and 77 per cent. of females. Apoplectiform attacks occurred in 41 per cent. of the malas, vertiginous attacks in 15½ per cent., epileptiform attacks in 10 per cent., and hysteriform attacks in 1 per cent. Among the females apoplectiform attacks occurred in 57 per cent., vertiginous attacks in 5½ per cent., epileptiform attacks in 5½ per cent, and hysteriform attacks in 4 per cent.

[8] Med. Rec., April 11, 1903. [9] Amer. Jour. of Med. Sciences, May, 1903. [10] Archives de Neurologie, Sept., 1902.

*(To be continued.)*

